# The role of UGT1A1 (c.-3279 T > G) gene polymorphisms in neonatal hyperbilirubinemia susceptibility

**DOI:** 10.1186/s12881-020-01155-2

**Published:** 2020-11-06

**Authors:** Zijin Li, Li Song, Lihong Hao

**Affiliations:** 1 Department of Internal Medicine, Tianjin Children’s Hospital, Tianjin, 300074 China; 2 Department of Neonatology, Tianjin Children’s Hospital, No. 238 Longyan Road, Beichen district, Tianjin, 300074 China

**Keywords:** UGT1A1, Neonatal hyperbilirubinemia, Neonatal jaundice, C.-3279 T > G, Polymorphism

## Abstract

**Background:**

Neonatal hyperbilirubinemia (NNH) is a common disease in newborns. This research study aimed to assess the associations between uridine diphospho-glucuronate-glucuronosyltransferase 1A1 (UGT1A1, c.-3279 T > G) polymorphisms and NNH risk.

**Methods:**

We searched PubMed, the Cochrane Library, and the Embase electronic databases. All published eligible studies before July 1, 2019, were searched for this meta-analysis.

**Results:**

We identified 7 independent studies including 1560 cases. The data showed that in the general population, compared with the GT + GG vs TT and GG vs TT, c.-3279 T > G (rs4124874) was significantly related to a higher NNH risk (GG vs TT: OR = 1.865, 95% CI: 1.031–3.373, *P* = 0.039; GT + GG vs TT: OR = 1.331, 95% CI: 1.055–1.679, *P* = 0.016). Although not statistically significant, the data showed that c.3279 T > G had a tendency to be associated with NNH under the allele model and GG vs GT + TT in the overall population (G vs T: OR = 1.288, 95% CI: 0.982–1.689, *P* = 0.067; GG vs TT + GT: OR = 1.583, 95% CI: 0.947–2.647, *P* = 0.080).

**Conclusion:**

The UGT1A1 gene c.-3279 T > G (rs4124874) polymorphism increased susceptibility to NNH, especially for the comparison of GT + GG vs TT and GG vs TT. In the future, we can use homozygous state of the UGT1A1 gene c.-3279 T > G (rs4124874) polymorphism for the diagnosis and screening of molecular biomarkers in NNH patients.

**Supplementary Information:**

The online version contains supplementary material available at 10.1186/s12881-020-01155-2.

## Background

Neonatal hyperbilirubinemia (NNH) is complex and involves multiple environmental and genetic risk factors [1–4]. Immature red blood cells and the liver and gastrointestinal tract are associated with benign transitions in hyperbilirubinemia [5]. Although considerable research effort has been focused on NNH, the pathophysiology of the disease remains incompletely understood. An increasing number of clinically relevant genetically identified diseases are recognized [6–8].

The uridine diphospho-glucuronate-glucuronosyltransferase 1A1 (UGT1A1) gene encodes bilirubin UDP glucuronosyltransferase, which is the enzyme responsible for bilirubin glucuronidation [9]. Recently, researchers have paid widespread attention to genetic factors that are particularly affected by UGT1A1 variants [4, 8, 10]. Approximately 113 UGT1A1 gene variants have been identified. In recent years, a c.-3279 T > G mutation (UGT1A1 * 60; rs4124874) was found in the UGT1A1 promoter, and some sequences in this promoter are related to NNH [11]. UGT1A1 * 60 is the nucleotide at the 3279 T-to-G substitution of UGT1A1, which is the reactive enhancer module phenobarbital. Nearly 60% of transcriptional activity is reduced at UGT1A1 * 60, so the increased risk of hyperbilirubinemia is inextricably linked to its presence [12].

In recent years, many studies have shown the role of c.-3279 T > G gene polymorphisms in NNH [8, 11, 13–16]. However, the results of these studies have not been determined. A single study of polymorphisms on NNH may not be sufficient to verify with a separate study. To show the impact of c.-3279 T > G gene polymorphisms on NNH risk, we identified eligible case-control studies and performed a meta-analysis.

## Methods

### Search strategy

The following keywords were used to research the articles in the Cochrane Library, PubMed and Embase electronic databases: “UGT1A1” OR “uridine diphospho-glucuronate-glucuronosyltransferase 1A1” OR “c.-3279 T > G” AND “polymorphism” OR “variant” OR “allele” OR “genotype” OR “neonatal hyperbilirubinemia” OR “neonatal jaundice” OR “hyperbilirubinemia”. All eligible studies were published before July 1, 2019. In addition, the reference lists were manually searched for additional studies. When multiple articles contained studies of the same population, only the most recent or complete study was used in the meta-analysis. The language of the publications was limited to English.

### Inclusion criteria and exclusion criteria

Articles had to meet the following criteria to be included in the meta-analysis: (1) The relationship between the genetic polymorphism of UGT1A1 (c.-3279 T > G) and the risk of NNH was evaluated; (2) a case-control study; and (3) the control group complies with Hardy-Weinberg equilibrium. The exclusion criteria strongly adhere to the following: (1) lack of valid raw data; (2) data had serious deviations; and (3) reproduction of publications.

### Data extraction

The data were independently evaluated by two reviewers. The difference was not resolved until the consensus of each item was reached. Information such as author name, year of publication, country of origin, ethnic origin, source of the control population, genotyping method, matching factors and adjustment factors, number of cases and controls were recorded in each item.

### Statistical analysis

This study was conducted independently by two researchers based on the information collected through reading relevant articles. Whether to include the studies in the meta-analysis was decided in strict accordance with the inclusion and exclusion criteria and discussion. ORs (odds ratios) and 95% CIs were used to estimate the relationships between the c.-3279 T > G gene polymorphism and NNH. To assess whether the results of the data set were heterogeneous, we used the Q test and I^2^. A heterogeneity *P* value below 0.05 was considered statistically significant for heterogeneity. The subgroup analysis of ethnicity was divided into Asians, Caucasians, and Africans. The random effects model was applied when the results of the heterogeneity test were *P* < 0.05 or I^2^ > 50%; otherwise, a fixed effect model was applied. Egger’s and Begg’s tests were used to detect publication bias, and P < 0.05 was considered a publication bias. A funnel graph and Egger’s test were used to display publication bias. All control groups were in Hardy-Weinberg equilibrium. STATA software was required for all meta-analyses (version 14.0; University City, Texas, USA). There were two sides for all tests.

## Results

### Study selection

**Fig. 1:**
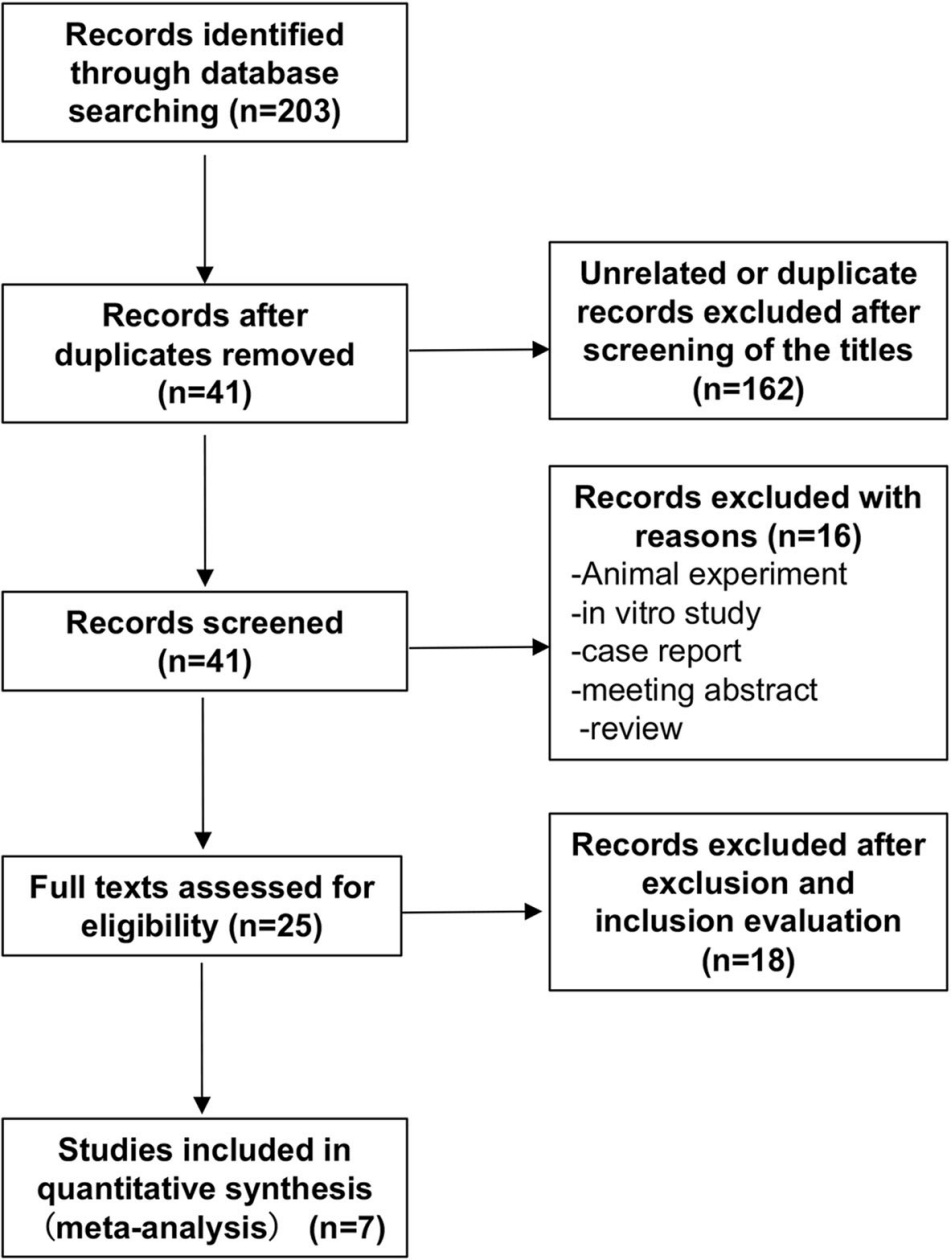
Flow diagram detailed procedures of selecting eligible studies

Figure 1 shows that we searched the bibliographic database and found 203 related records. After several rounds of screening, 25 articles were found that met the search criteria. After applying the inclusion and exclusion evaluation criteria to the articles, a total of 7 studies met the criteria [8, 11, 13–17].

### Study characteristics

Seven independent studies were identified, which included 1560 cases. Table 1 lists the main characteristics of all eligible studies. There are 7 case-control studies on c.-3279 T > G (rs4124874) [8, 11, 13–17]. The seven independent studies included 5 studies on Asian populations [8, 11, 14–16], 1 African study [17] and 1 Caucasian study [13].

**Table 1:** Basic information of the original articles included in this meta-analysis

Author	Ethnicity	Year	Study design	Methods	Case			Control		
					GG	AA	GA	GG	AA	GA
Watchko et al.	Caucasian	2009	HBC	IBS	41	35	77	89	74	135
Yusoff et al.	Asian	2010	HBC	PCR-RFLP	18	19	29	4	28	38
Tiwari et al.	Asian	2013	HBC	PCR-RFLP	21	35	44	26	28	46
Tiwari et al.	Asian	2014	HBC	PCR-RFLP	32	21	60	45	69	104
D’Silva et al.	Asian	2014	PBC	PCR-RFLP	46	25	55	52	61	68
Tomerak et al.	African	2016	HBC	PCR-RFLP	20	32	11	2	18	18
Yanagi et al.	Asian	2017	HBC	PCR-RFLP	0	3	1	1	16	13

*PCR—RFLP* Polymerase chain reaction—restriction fragment length polymorphism, *IBS* Internet-based system, *HBC* Hospital-based study, *PBC* Population-based study

### C.-3279 T > G (rs4124874)

**Fig. 2:**
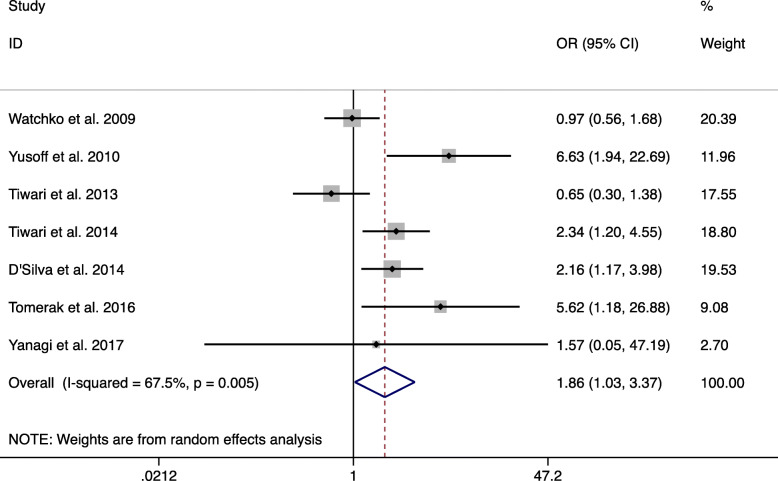
Random-effects meta-analysis on NNH risk and c.-3279 T > G (rs4124874) polymorphism in overall population (GG versus TT)

**Fig. 3:**
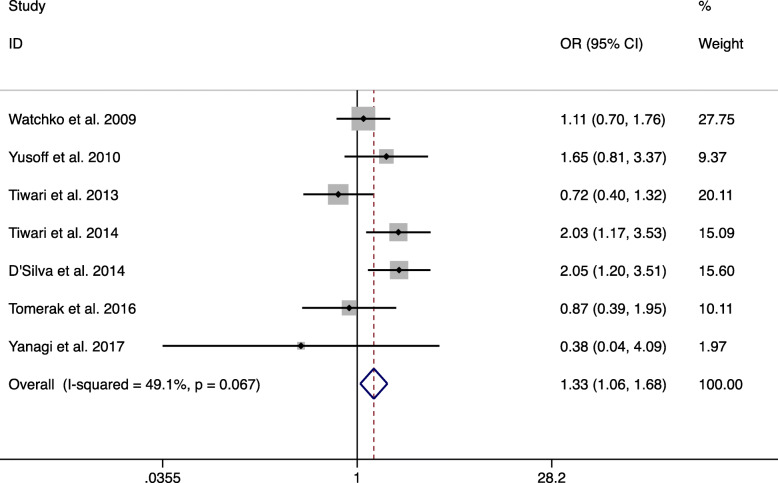
Fixed-effects meta-analysis on NNH risk and c.-3279 T > G (rs4124874) polymorphism under GT + GG vs TT in overall population (GT + GG vs TT)

For c.-3279 T > G (rs4124874), the main results are shown in Table 2. These data show that c.-3279 T > G (rs4124874) was significantly related to a higher NNH risk in the overall population (GG vs TT and GT + GG vs TT) (GG vs TT: OR = 1.865, 95% CI: 1.031–3.373, *P* = 0.039, Fig. 2; GT + GG vs TT: OR = 1.331, 95% CI: 1.055–1.679, *P* = 0.016, Fig. 3). Although not statistically significant, the data showed that c.-3279 T > G had a tendency to be associated with NNH under the allele model and GG vs GT + TT in the overall population (G vs T: OR = 1.288, 95% CI: 0.982–1.689, *P* = 0.067; GG vs TT + GT: OR = 1.583, 95% CI: 0.947–2.647, *P* = 0.080).

**Table 2:** Summary ORs (95% CI) of UGT1A1 (c.-3279 T > G) gene polymorphisms and neonatal hyperbilirubinemia risk

Genetic model	Subgroup analysis	No. of studies	OR (95% CI)	P	I^2^%	P (Q test)	Egger’s test	Begg’s test
G vs T	Total	7	1.288 (0.982–1.689)	0.067	63.9	0.011	0.766	1.000
	Asian	5	1.334 (0.939–1.896)	0.108	66.3	0.018		
	African	1	1.669 (0.907–3.072)	0.100	0	1.000		
	Caucasian	1	0.978 (0.742–1.289)	0.874	0	1.000		
GG vs TT	Total	7	1.865 (1.031–3.373)	**0.039**	67.5	0.005	0.203	0.368
	Asian	5	1.958 (0.946–4.052)	0.070	67.3	0.016		
	African	1	5.625 (1.177–26.877)	0.030	0	1.000		
	Caucasian	1	0.974 (0.564–1.682)	0.925	0	1.000		
GG vs GT	Total	7	1.557 (0.883–2.745)	0.126	71.3	0.002	0.057	0.072
	Asian	7	1.365 (0.806–2.314)	0.247	51.5	0.083		
	African	1	16.364 (3.188–83.993)	0.001	0	1.000		
	Caucasian	1	0.808 (0.508–1.284)	0.367	0	1.000		
GT vs TT	Total	7	1.100 (0.720–1.681)	0.660	58.6	0.024	0.331	0.368
	Asian	5	1.322 (0.849–2.057)	0.216	43.3	0.133		
	African	1	0.344 (0.133–0.886)	0.027	0	1.000		
	Caucasian	1	1.206 (0.739–1.968)	0.454	0	1.000		
GT + GG vs TT	Total	7	1.331 (1.055–1.679)	**0.016**	49.1	0.067	0.603	0.764
	Asian	5	1.427 (0.875–2.326)	0.154	58	0.049		
	African	1	0.872 (0.389–1.952)	0.739	0	1.000		
	Caucasian	1	1.114 (0.703–1.764)	0.646	0	1.000		
GG vs TT + GT	Total	7	1.583 (0.947–2.647)	0.080	69.5	0.003	0.084	0.072
	Asian	5	1.549 (0.894–2.685)	0.119	60.1	0.040		
	African	1	8.372 (1.832–38.260)	0.006	0	1.000		
	Caucasian	1	0.860 (0.556–1.329)	0.496	0	1.000		
GT vs TT + GG	Total	7	0.903 (0.631–1.293)	0.578	59.2	0.023	0.061	0.133
	Asian	5	1.062 (0.821–1.374)	0.648	0	0.434		
	African	1	0.235 (0.095–0.584)	0.002	0	1.000		
	Caucasian	1	1.223 (0.828–1.808)	0.312	0	1.000		

*OR* Odds ratio, *CI* Confidence interval, *vs* versus, *P (Q test)* P value of Q test for heterogeneity test

### Subgroup analysis

**Fig. 4:**
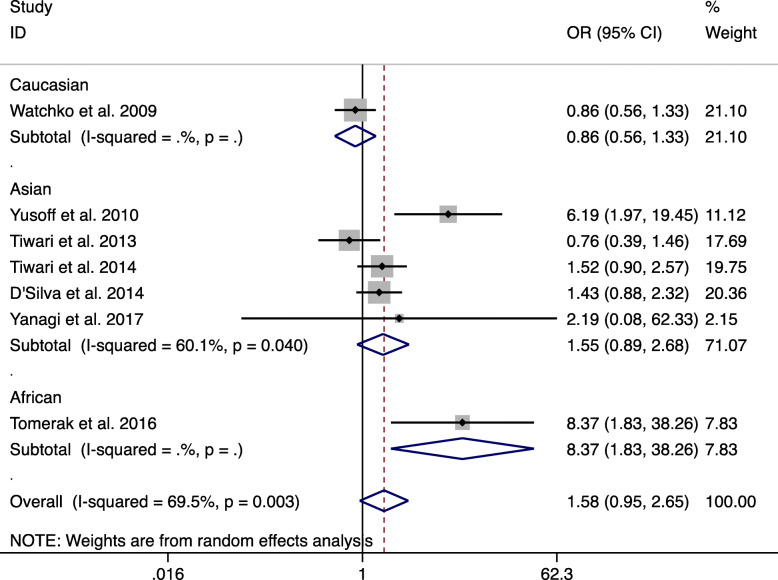
Random-effects meta-analysis on NNH risk and c.-3279 T > G (rs4124874) polymorphism under GG vs GT + TT in African population (GG vs TT + GT)

The subgroup analysis in this paper is mainly based on ethnic differences, and Asians, Caucasians and Africans were grouped separately. The prevalence of the G and T alleles among different races is shown in Supplemental Table S1. A correlation with a higher NNH risk was found in the subgroup analysis, which was compared with GG vs TT and GG vs GT + TT in the African population (GG vs TT: OR = 5.625, 95% CI: 1.177–26.877, *P* = 0.03; GG vs TT + GT: OR = 8.372, 95% CI: 1.832–38.260, *P* = 0.006, Fig. 4). However, an association with a lower NNH risk was detected in the comparison of GT vs TT and GT vs TT + GG in the African population (GT vs TT: OR = 0.344, 95% CI: 0.133–0.886, *P* = 0.027; GT vs TT + GG: OR = 0.235, 95% CI: 0.095–0.584, *P* = 0.002).

### Sensitivity analyses

One-way sensitivity analysis was performed on the data involved in this meta-analysis. Each single study of the meta-analysis was deleted each time to reflect the overall impact on the individual data sets, and the corresponding combined results were not substantially changed.

### Publication bias

**Fig. 5:**
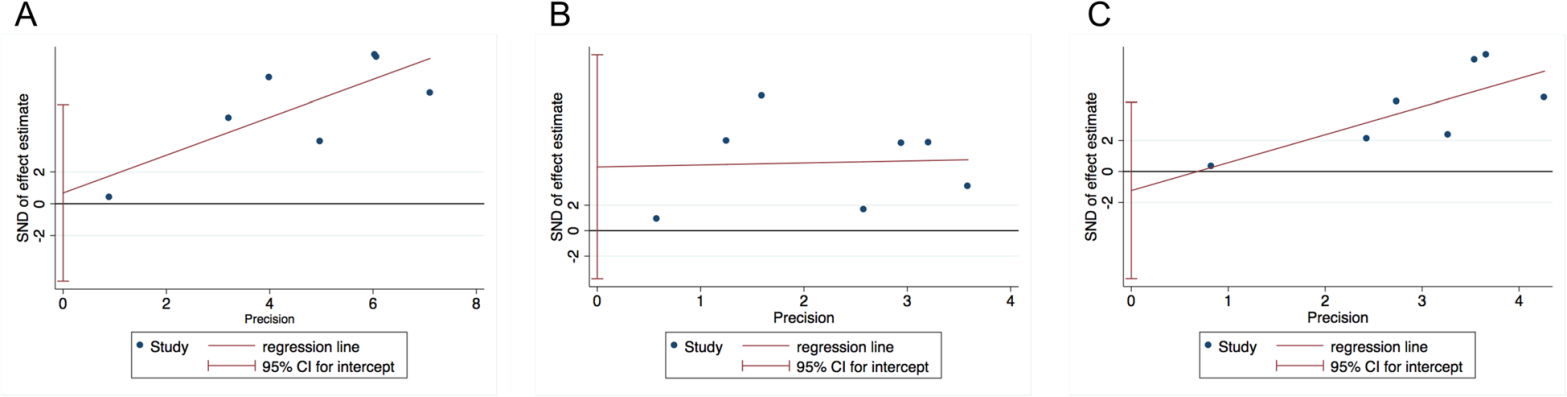
The Egger linear regression test for c.-3279 T > G (rs4124874) compared with the (**a**) allele model, **b** the contrast of GG vs TT and **c** GT + GG vs TT to show publication bias

Publication bias was assessed by Begg’s test and Egger’s test (Table 2). A graph of Egger’s linear regression test was compared to the allele model (Fig. 5a). The contrast of GG vs TT (Fig. 5b) and GT + GG vs TT (Fig. 5c) for c.-3279 T > G gene polymorphisms was evaluated to intuitively show publication bias. In addition, evidence of publication bias could not be found in any other analysis in various comparative models.

## Discussion

This study showed that the modifiable risk of neonatal hyperbilirubinemia includes multiple genetic and clinical risk factors. Genes that can exacerbate mutations have lower transcriptional activity in the first few days of life because their liver UGT1A1 is natural and immature. UGT1A1 is regulated in a developmental manner. Its activity can increase from 0.1% at 17–30 weeks of gestation to 1% at 30–40 weeks, reaching the adult level only 14 weeks after birth [18]. It has been shown that UGT1A1 (c.-3279 T > G) gene polymorphisms might have an effect on NNH risk [15, 17, 19]. However, a single study of the relationship between NNH and genes is controversial. Some studies reported that the UGT1A1 (c.-3279 T > G) gene polymorphisms did not have a role in NNH risk [13, 14]. The problem of low statistical power in a single study can be solved by meta-analysis, and stronger conclusions can be drawn. UGT1A1 gene polymorphisms are associated with many diseases and have been confirmed by meta-analysis [20–23].

Moreover, a meta-analysis confirmed that the TATA box polymorphism and c.211G > A (Gly71Arg) of the UGT1A1 gene polymorphism are associated with NNH [24–27]. Therefore, we evaluated the role of UGT1A1 in this meta-analysis (c.-3279 T > G) gene polymorphism in neonatal hyperbilirubinemia susceptibility. Seven case-control studies contained in this meta-analysis showed that the c.-3279 T > G (rs4124874) UGT1A1 gene polymorphism was related to the risk of GG vs. TT and GT + GG vs TT in the overall population. Although not statistically significant, the data showed that c.-3279 T > G has a tendency to be associated with NNH under the allele model and GG vs GT + TT in the overall population.

One disturbing problem is the analysis of population groups, which is often not very reliable in the presence of evidence of disease, which indicates that the environment and race affect genetic background differently [28]. Simultaneously, although different populations have the same polymorphisms in racial subgroup analysis, susceptibility plays different roles in the population. We analyzed the prevalence of the G and T alleles between different races. Differences between different races have little effect on the overall results. However, there are some differences between the African population and the Asian or Caucasian populations in this study. Therefore, it should be noted that the results of the subgroup analysis of the African population need to be further confirmed. The number of African studies included is small, and more literature should be included in the future to verify this result.

This meta-analysis has some limitations. First, heterogeneity may have interfered with the results of this meta-analysis. Nonetheless, we use specific research standards to strictly perform data extraction and analysis to minimize this possibility. In addition, published studies were included in this meta-analysis, and results with nonsignificant or negative results might not be published, so the possibility of bias may be increased in publication. Finally, our results have not been adjusted. If more research data can be obtained, a more accurate analysis could be performed. Other variables can be used, including race, age, family history, environmental factors and lifestyle [29–32].

Although there were limitations in this meta-analysis, we found that the UGT1A1 gene c.-3279 T > G (rs4124874) polymorphism is associated with NNH risk. Therefore, it can be used as a screening index for NNH. In the future, we should consider using a larger sample size to confirm our results. In addition, the effects of gene-environment and gene-gene interactions must be researched in the future. An in-depth analysis of these factors provides a more complete understanding and more reliable results of the linkage between the UGT1A1 gene c.-3279 T > G (rs4124874) polymorphism and the risk of NNH.

## Conclusion

In summary, the UGT1A1 gene c.-3279 T > G (rs4124874) polymorphism increased susceptibility to NNH, especially for the comparison of GT + GG vs TT and GG vs TT. In the future, we can use homozygous state of the UGT1A1 gene c.-3279 T > G (rs4124874) polymorphism for the diagnosis and screening of molecular biomarkers in NNH patients.

## Supplementary Information

**Additional file 1 **: **Supplemental Table S1.** The prevalence of G and T allele among different races

## Data Availability

The datasets used and analyzed in the present study are available from the corresponding author upon reasonable request.

## Abbreviations

NNH: Neonatal hyperbilirubinemia
UGT1A1: Uridine diphospho-glucuronate-glucuronosyltransferase 1A1
OR: Odds ratio
CI: Confidence interval
