# Supplementary material for: The role of UGT1A1 (c.-3279 T > G) gene polymorphisms in neonatal hyperbilirubinemia susceptibility
Source: BMC Med Genet. 2020 Nov 6;21:218. doi: 10.1186/s12881-020-01155-2 (PMC7648392; doi:10.1186/s12881-020-01155-2)
Supplement: Supplementary file 1 — Additional file 1 : Supplemental Table S1. The prevalence of G and T allele among different races [file 12881_2020_1155_MOESM1_ESM.docx]

**Supplemental Table S1.** The prevalence of G and T allele among different races

| Ethnicity | Case (%) | | Control (%) | |
| --- | --- | --- | --- | --- |
|  | G | T | G | T |
| Asian | 423 (51.7) | 395 (48.3) | 525 (43.8) | 673 (56.2) |
| Caucasian | 159 (52.0) | 147 (48.0) | 313 (52.5) | 283 (47.5) |
| African | 51 (40.5) | 75 (59.5) | 22 (28.9) | 54 (71.1) |
| Total | 633 (50.6) | 617 (49.4) | 860 (46.0) | 1010 (54.0) |
